# Highly controlled multiplex electrospinning

**DOI:** 10.1186/s11671-024-04035-3

**Published:** 2024-06-06

**Authors:** Isaac C. Gilfeather, Harold W. Pearson-Nadal, Jessica M. Andriolo, Jack L. Skinner

**Affiliations:** 1 Mechanical Engineering Department, Montana Technological University, 1300 West Park St., Butte, MT 59701 USA; 2 Montana Tech Nanotechnology Laboratory, Montana Technological University, 1300 West Park St., Butte, MT 59701 USA; 3 Materials Science Ph.D. Program, Montana Technological University, 1300 West Park St., Butte, MT 59701 USA

**Keywords:** Electrospinning, Polymers, Electrostatics, Control systems, Nanofibers, Flexible electronics

## Abstract

Applications of electrospinning (ES) range from fabrication of biomedical devices and tissue regeneration scaffolds to light manipulation and energy conversion, and even to deposition of materials that act as growth platforms for nanoscale catalysis. One major limitation to wide adoption of ES is stochastic fiber deposition resulting from the chaotic motion of the polymer stream as is approaches the deposition surface. In the past, fabrication of structures or materials with precisely determined mesoscale morphology has been accomplished through modification of electrode shape, use of multi-dimensional electrodes or pins, deposition onto weaving looms, hand-held electrospinning devices that allow the user to guide deposition, or electric field manipulation by lensing elements or apertures. In this work, we demonstrate an ES system that contains multiple high voltage power supplies that are independently controlled through a control algorithm implemented in LabVIEW. The end result is what we term “multiplex ES” where multiple independently controlled high-voltage signals are combined by the ES fiber to result in unique deposition control. COMSOL Multiphysics® software was used to model the electric field produced in this novel ES system. Using the multi-power supply system, we demonstrate fabrication of woven fiber materials that do not require complex deposition surfaces. Time-varied sinusoidal wave inputs were used to create electrospun torus shapes. The outer diameter of the tori was found, through parametric analysis, to be rather insensitive to frequency used during deposition, while inner diameter was inversely related to frequency, resulting in overall width of the tori increasing with frequency. Multiplex ES has a high-frequency cutoff based on the time response of the high voltage electrical circuit. These time constants were measured and minimized through the addition of parallel resistors that decreased impedance of the system and improved the high-frequency cutoff by up to 63%.

## Introduction

Electrospinning (ES) fabrication was first observed in 1897 [1] followed by a series of patents granted for textile applications. In 1969, a publication by Taylor [2] resulted in research that utilized ES fabrication for many applications that sought to make polymer materials with micro to nano-scale features that exhibited high surface-area-to-volume ratios. Since then, ES has been used to fabricate fuel cells, generators, and provide photocatalytic surfaces [3]. As well as to prevent degradation of perovskite solar cell layers [4–7] and to pattern nanoscale polarizers via lithography [8]. Biomedical applications of electrospun materials include enzyme immobilization, sensors, tissue engineering, wound healing [9], and drug delivery [10–12]. ES fiber materials have also been used for the creation of nanomaterials that range in application from energy conversion to medicine and that exhibit desirable material properties, such as high strength or modulus [13].

ES fabrication requires delivery of a solvent-dissolved [14–16] or melted [17] liquid polymer into a high strength electrostatic field that exists between a metallic spinneret and a collection surface. Once the liquid polymer reaches end of the spinneret, the electrostatic field causes surface charge buildup at the surface of the polymer bead at the end of the spinneret. At critical value, the polymer bead is deformed into a Taylor cone [2]. At the tip of the Taylor cone, a micro or nano-scale polymer jet is pulled by electrostatic force toward the deposition surface, resulting in deposition of polymer fibers or beads. During flight, the polymer jet experiences a chaotic phase, whereby, solvent evaporation occurs [18]. The force required to initiate ES is described by the following formula:

1 $$\begin{array}{*{20}c} {F_{es} = \frac{{\varepsilon_{r} \varepsilon_{0} }}{{2d^{2} }}V^{2} A} \\ \end{array}$$

where permittivity is represented by $\varepsilon_{r}$ (relative) and $\varepsilon_{0}$ (in a vacuum), $A$ is the area of the collection plate, $V$ is the applied voltage, and $d$ is the separation distance between spinneret and collection surface [19].

The breadth of materials that ES has enabled is far reaching and relevant in application from fundamental chemistry and materials synthesis to applied use in industry. The span of applicable uses for ES has led to iterations of ES equipment that accommodate implementation for fabrication of specialized materials. Melt ES, for example, allows the user to avoid the use of solvents during the process [17]. Other iterations involve alteration of the deposition surface to produce aligned structures that are beneficial for enhanced charge transport [20, 21], production of polarized light emission [22, 23], improved absorption and photovoltaic properties [24, 25], and crystal properties beneficial for optoelectronics among other applications [26–29]. Alignment is also relevant to the biomedical industry to provide a scaffold for directional cell growth [30] and guided cell differentiation [31]. Alignment of polymer fibers can be accomplished through the use of rotating collector drums [32, 33] parallel gap electrodes [8, 34], or counter electrodes [35]. The electric field that provides electrostatic force for polymer deposition has also been manipulated to guide fiber deposition and material spot size [19, 36, 37]. Passive methods for electric field manipulation include using copper rings as lensing elements to dampen chaotic motion [38] and use of aperture plates to reduce resulting fiber mat spot size [36, 39]. Researchers have also accomplished miniaturization of the ES system and added configuration modifications that allow ES systems to be handheld and deposit onto any surface regardless of charge [37, 40, 41].

In this work, we present an iteration of ES fabrication that enables precisely controlled deposition of electrospun fibers by use of multiple high voltage power supplies, each linked to separate electrodes and controlled by a wave generator. Sinusoidal control (input) signals with appropriate phase lag were generated in LabVIEW to control fiber deposition in two dimensions, which resulted in woven polymer fabrics and complex shapes. Woven polymer fabrics are desirable for strength, dimension, flexibility, porosity, elongation, and failure strength in multiple directions [42] in addition to enabling long-term drug release [43] and tissue mimicry [44]. The production of woven electrospun materials is performed through the use of novel deposition surfaces, such as weaving-looms [43] or rotating collectors with conducting tines [45]. In this work, we demonstrate multiplex ES with multiple independently-controlled high-voltage power supplies to create woven polymer fabrics on flat, non-complex surfaces with precise control rather than random attachment and deposition onto complicated, moving surfaces. Our novel process enables coating of objects or materials that are placed onto the flat deposition surface that are not feasible for loom or conducting tine deposition substrates. Time-varied sinusoidal wave inputs were also used to demonstrate deposition of electrospun torus shapes that have not been demonstrated with other highly-controlled ES systems. Using parametric analysis, predictable torus dimensions can be achieved. Demonstration of torus deposition among other complex morphologies provides examples of the highly controlled structures made possible by the multiplex ES system. Deposition of complex polymer morphologies expands applicability of this versatile and economically feasible manufacturing method for producing flexible materials that can wrap around bone, coat a sharp corner, or be shaped to slide into a non-linear crevice to enable novel functional materials. For multiplex ES, the time constant that dictates the high-voltage high frequency cutoff is also reduces through the reduction of electrical impedance. Parallel resistors added to the high voltage circuit reduced the circuit impedance, lowering the time constant and further demonstrating the high level of fiber deposition control multiplex ES can provide.

## Methods

### Component selection and system assembly

**Fig. 1:**
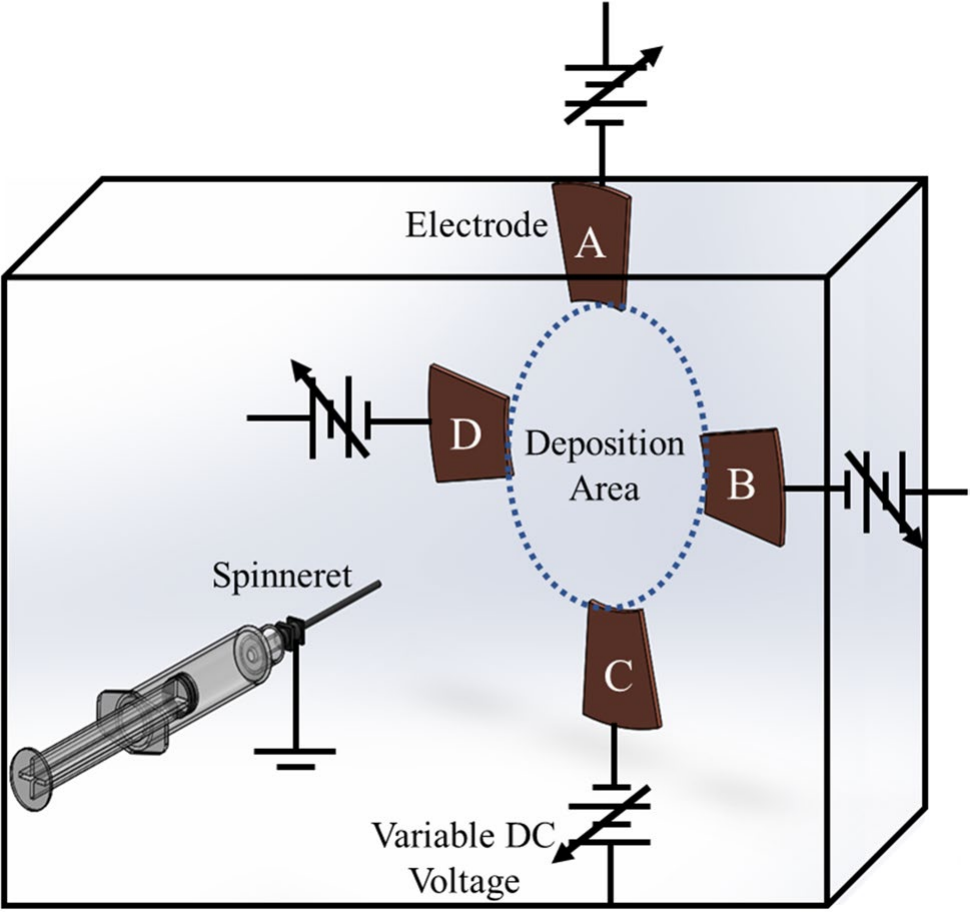
Graphical representation of multiplex ES system. The multiplex ES system contains four electrodes (**A**–**D**) and a spinneret, each connected to an independently-controlled power supply. Each voltage input to the system is controlled by a National Instruments DAQ and controlled by an algorithm implemented in LabVIEW. Independent control over each electrode enables deposition of complex structures

The multiplex ES system (Fig. 1) was assembled inside an isolation box assembled from 0.32-cm-thick acrylic sheets and equipped with a momentary safety switch. The safety switch was integrated into the system via LabVIEW and programmed to force the connected data acquisition system (DAQ) output to 0 kV should the door be opened. The DAQ systems used for this work were USB-6008 and USB-6009 12-bit resolution National Instruments digital acquisition DAQs that provided the input control signals, either direct current (DC) and alternating current (AC), to the high-voltage power supplies. Aluminum tape placed at the edges of the acrylic sheets of the isolation box was grounded to prevent charge accumulation on the surfaces of the box.

At one side of the isolation box, a photopolymer holding stand was used to hold the needle in place and connected to high voltage via contact with a metal pogo pin. Holder pieces were fabricated with a Formlabs © Form2 405 nm SLA resin 3D printer using Formlabs© standard photopolymer resin followed by an ultraviolet curing step. The photopolymer was used in place of fused deposition modelling printing to provide a dense, solid part without air spaces for an enhanced dielectric constant and electrostatic field shielding. At the other end of the isolation box, four electrodes were held in place with the same photopolymer to keep each electrode connected to high voltage by metal pogo pin. The four deposition electrodes used were cut from 0.04-cm-thick Cu sheets. Each electrode was connected to a 20 kV (1 mA maximum) high-voltage power supply from ESDEMC Technology LLC and was controlled by a National Instruments DAQ system. Feedback from the four power supplies was monitored by a Tektronix TDS 2004C oscilloscope.

### Polymer preparation

Polycaprolactone (PCL) was purchased from Sigma Aldrich (80 k MW) was used because of its suitability for ES and biocompatibility. Compared to other polymers we have used in the lab, PCL has provided us with the most consistent morphological results when deposited by ES and provides a hydrophobic structure that stays in tact in any humidity and for electron microscopy preparation and imaging. PCL was prepared at 9 wt% in 2, 2, 2-trifluoroethanol (TFE, Sigma Aldrich) by stirring on a hot plate at 90 °C.

### Electrospinning parameters

The grounded spinneret used during ES was 22 gauge, and separation distances ranged from 10 to 20 cm, while polymer flow rate ranged between 0.25 and 0.45 mL/hr. Input voltage from the DAQ ranged from 0 to 5 V, which resulted in a 0–20 kV output from the power supplies (scaled linearly) to the electrodes. Multiplex ES was performed in a laboratory held at 27 °C and 25% relative humidity. Higher temperature during fiber formation has been shown to increase solvent evaporation rate and polymer viscosity decreased with increasing temperature [46]. During ES, the solvent-dissolved polymer is electrified, thereby creating electrostatic repulsion among the surface charges that feature the same polarity [47]. To improve repeatability, such parameters should be controlled during ES.

### COMSOL modeling of the electrostatic field

The electric field of the multiplex ES system was modelled using COMSOL Multiphysics® software. The model was based on solutions to the Poisson’s equation and mapped the electric field in three dimensions, which allowed conceptualization of the system geometry and how system configuration affected electrostatic forces that act on the polymer during ES.

### Microscopy

A Hitachi S-4500 field emission scanning electron microscope (SEM) was used to image resultant ES mats. Preparation for SEM involved adhering the mats to aluminum stubs by carbon tape and gold coating the samples in a Denton Desktop unit for 1 min. Secondary imaging was collected on a Keyence VHX-5000 digital light microscope.

### ImageJ analysis of electrospun tori

Electrospun tori were imaged with a camera that had 82.4 pixel/cm average resolution at a distance of 30.5 cm. Camera images were then thresholded in ImageJ software. To define the pixel boundaries, a threshold of 95/255 was applied to the images, allowing the edges of the tori mats and the background of the image to be distinguished and the dimensions of the tori mats to be measured.

## Results

### Electrostatic force models for multiplex ES

**Fig. 2:**
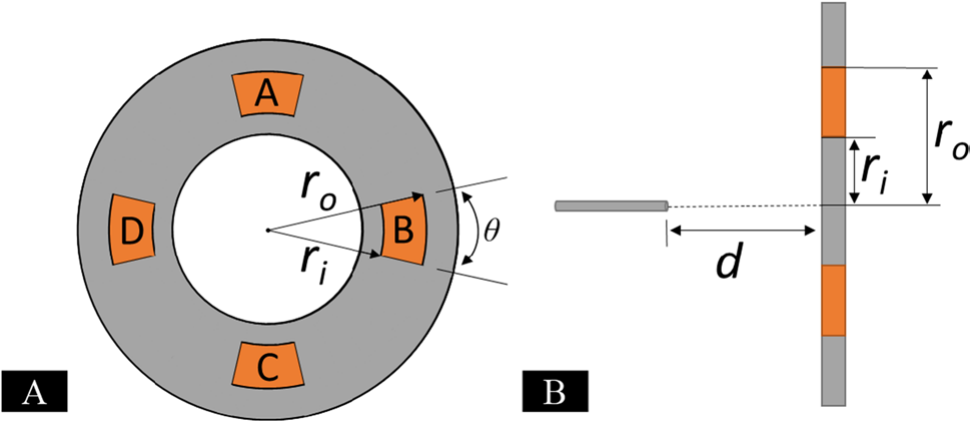
**A** Graphical representation of the electrode setup for the multiplex ES system showing the symmetrically spaced electrodes used. **B** Incorporation of the electrode shape shown in part **A** along with the separation distance of the needle to the deposition surface

The electrostatic force for the multiplex ES system is represented by Eq. 1, where permittivity is represented by $\varepsilon_{r}$ (relative) and $\varepsilon_{0}$ (in a vacuum), $A$ is the area of the collection plate, $V$ is the applied voltage, and $d$ is the separation distance between spinneret and collection surface. In the four-electrode setup, the four electrodes were symmetrically spaced apart from the spinneret with shapes defined by circular sectors of angle *θ*, inner radius *r*_*i*,_ and outer radius *r*_*o*_ (Fig. 2).

Integration along the surface of the electrode allows the magnitude of the electrostatic force $\left( {F_{es} } \right)$ between individual electrodes to be calculated as follows:

2 $$dF_{es} = \frac{{\varepsilon_{r} \varepsilon_{0} }}{{2h^{2} }}V^{2} dA$$

3 $$\int dF_{es} = \int \frac{{\varepsilon_{r} \varepsilon_{0} }}{{2h^{2} }}V^{2} dA$$

4 $$dA = rdrd\theta$$

5 $$h = \sqrt {r^{2} + d^{2} }$$

6 $$F_{es} = \frac{{\varepsilon_{r} \varepsilon_{0} }}{4}V^{2} \theta \left[ {\log \left( {r_{o}^{2} + d^{2} } \right) - \log \left( {r_{i}^{2} + d^{2} } \right)} \right]$$

where permittivity is represented by $\varepsilon_{r}$(relative) and $\varepsilon_{0}$ (in a vacuum), sector angle *θ*, inner radius *r*_*i*_ and outer radius *r*_*o*_,* V* is the applied voltage, and *d* is the separation distance between spinneret and collection surface.

Since the electrode geometry was symmetric with respect to the spinneret, the location of nanofiber deposition becomes proportional to the balanced amount of electrostatic force directed in the direction of each electrode. The summation of the electrostatic forces in each direction is what ultimately determines the driving force causing deposition and the most likely deposition location of the nanofiber.

**Fig. 3:**
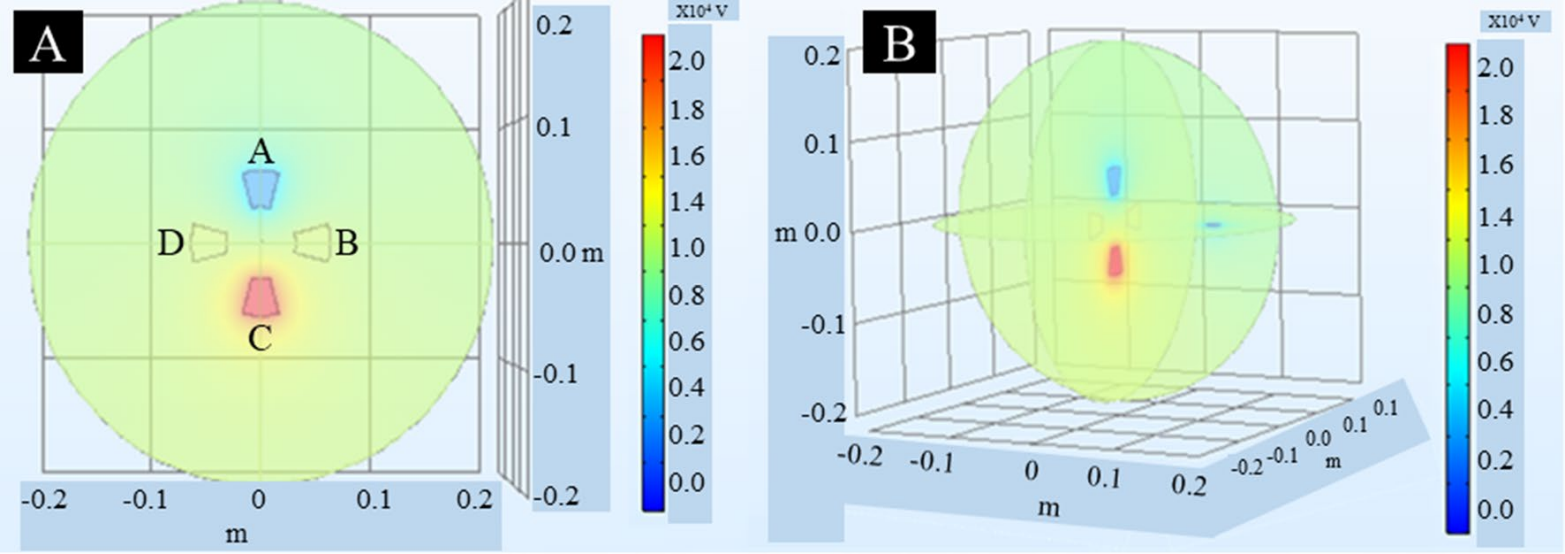
COMSOL Multiphysics® model showing electrostatic field strength within the multiplex ES system. **A** The generated model shows four electrodes placed equidistant from each other. During acquisition of the model, the high-voltage signal was supplied to electrode C as shown. **B** The generated model shows the electrodes from **A** with respect to the ES spinneret. All electrodes were placed equidistant from the spinneret in the multiplex ES system

The electrostatic field within the multiplex ES system was modelled in COMSOL Multiphysics® software. Values for the voltage at each electrode were consistent with the input control signals generated in LabVIEW, amplified by the high-voltage power supplies, and supplied to the electrodes (Fig. 3A). The spinneret was grounded (0 V) during the experiment and is visible in blue and surrounded with a smooth transition to the high voltage environment (Fig. 3B).

### Deposition of woven electrospun mats by multiplex ES

**Fig. 4:**
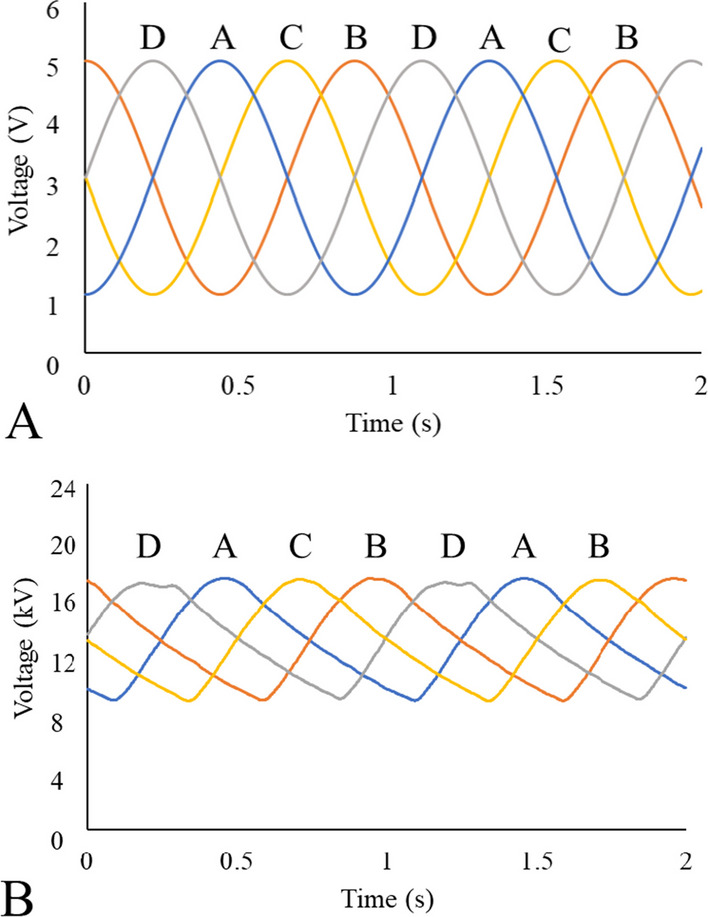
**A** Input signals generated in LabVIEW and fed through the DAQ. **B** Output signals were measured with an oscilloscope to be 4 kX that of the input signals. Variation between the signals is due to the response time of the high voltage power supplies

Multiple, time-varied signal inputs were generated in LabVIEW and used to control the voltage on the power supplies, thereby directing fiber deposition. The sinusoidal input used consisted of a 2 V amplitude, 3 V offset, 1 Hz frequency, and 90° phase shift between each signal, amplified by 4 kX by the high voltage power supplies used. Generated input and measured output signals are shown in Fig. 4.

**Fig. 5:**
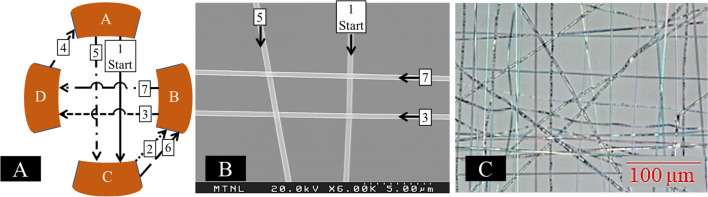
**A** Graphical representation of fiber deposition during multiplex ES of woven fiber mats. During deposition, fiber deposition follows the electrode exhibiting the highest voltage (strongest electrostatic force). In instances 2, 4, and 6, the polymer jet jumps exterior to the center woven mat in order to avoid disrupting the woven material and enabling true weaving of the fibers to occur. **B** A representative SEM micrograph that demonstration the deposited weave pattern exactly. **C** A digital light microscopy image shows the bulk three-dimensional configuration of the woven ES mats

Using this input, woven PCL fiber mats were produced by the pattern shown in Fig. 5A. The woven pattern is represented on an electron micrograph in Fig. 5B. During deposition, the polymer jet is directed toward the direction of the maximum electrostatic field, which changes with time according to the control scheme. Under the wave input conditions used, the high strength electrostatic force is alternated between the four electrodes. In this system, polymer was first deposited onto electrode A, followed by electrode C. From C, the polymer jet is directed to electrode B, still following the high strength field but avoiding the center mat. From electrode B, the polymer fibers are directed across to electrode D, then to A (avoiding the center mat), and then onto C, to B (avoiding the center mat), and back to electrode D. This pattern results in truly woven polymer mats fabricated in the central area, between electrodes (Fig. 5C).

### Parametric analysis of tori deposited by multiplex ES

**Fig. 6:**
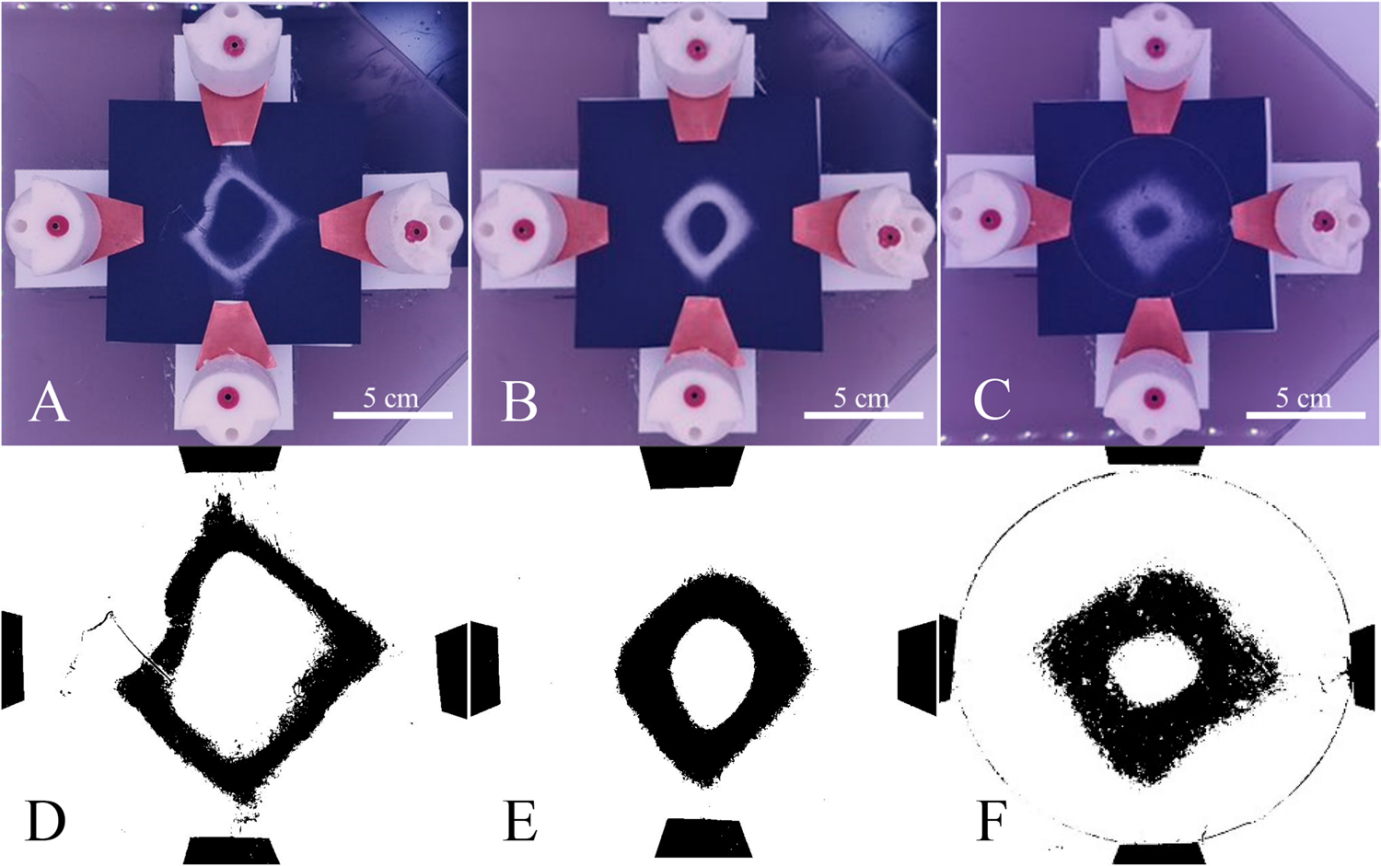
**A**–**C** Images of electrospun fiber tori fabricated using multiplex ES. Electrospun tori were removed from the system and placed in a light box prior to acquisition of images, which were thresholded with ImageJ. **D**–**F** Corresponding images (top to bottom) showing the tori fiber mats after a threshold had been applied in ImageJ. Tori and images were collected in triplicate, and dimensions from these images were used to provide mathematical understanding of the fiber mat that would result when specific ES parameters were used

**Fig. 7:**
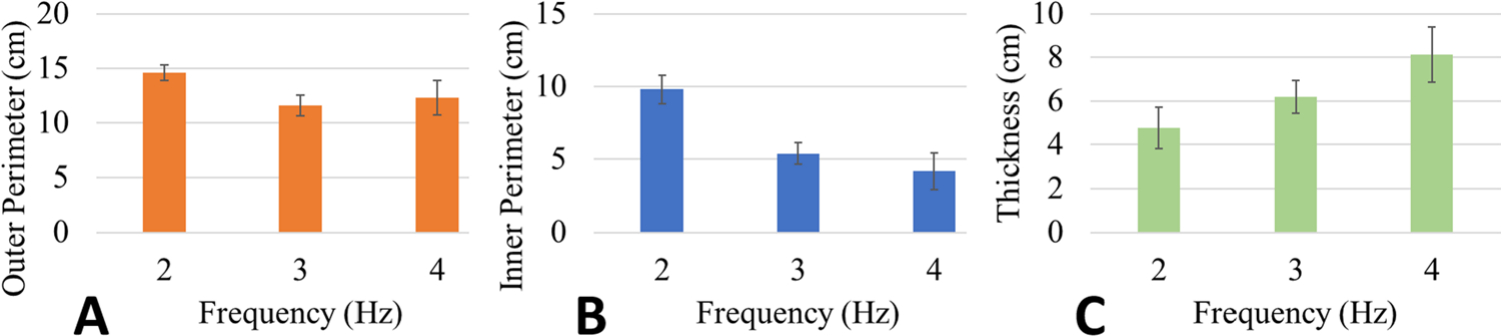
Parametric analysis of electrospun tori. **A** Outer thickness was not significantly different between electrospun samples controlled by a sinusoidal input of 2, 3, or 4 Hz (*p* = 0.088).** B** The inner diameter of the tori decreased with increasing frequency (*p* < 0.015).** C** Thickness of the fiber mat increased with increasing frequency, with the thickest tori being produced at 4 Hz (*p* ≤ 0.05)

Parametric analysis has been used previously to generate mathematical understanding of the relationship between ES parameters and the resultant fiber mat spot size [19, 37]. The multiplex ES device enables deposition of fiber mats with features that exhibit higher complexity. Parametric analysis of frequency used during ES was used to provide mathematical understanding of the resultant fiber dimensions when multiplex ES was used to deposit torus structures (Fig. 6). During ES, all parameters were held constant with exception of frequency. Frequencies of 2, 3, and 4 Hz were used during these studies, and tori were deposited and imaged in triplicate (Fig. 6A–C). ImageJ was then used to apply a threshold to the fiber tori structures (Fig. 6D–F), and resultant dimensions were compared. Results showed that the outer dimensions of the tori were rather insensitive to alterations to frequency (*p* = 0.088, Fig. 7A). However, the inner diameter of the tori varied inversely with frequency of the applied electrostatic field described in the previous section (Fig. 7B). In all comparisons, mats deposited at 2, 3, and 4 Hz exhibited inner diameters that varied significantly (*p* < 0.015 or less). The alteration to the inner diameter of the tori ultimately resulted in alterations to the overall thickness of the structures (Fig. 7Cp, p = 0.05 or less).

### Intricate designs enabled by multiplex ES

**Fig. 8:**
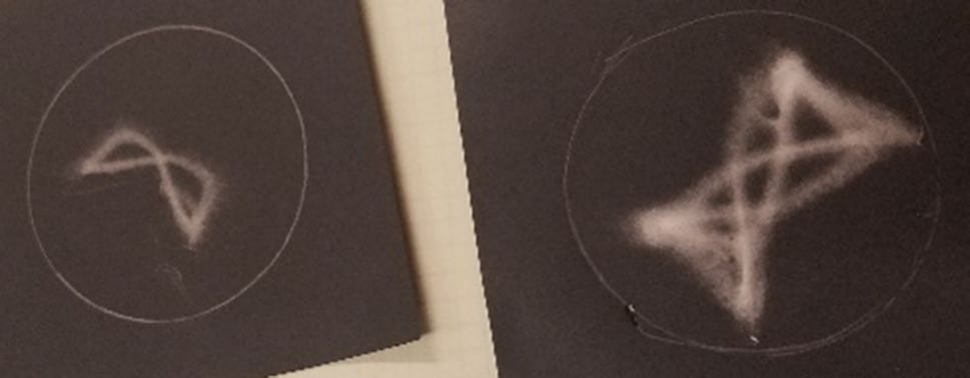
Photographs of looped ring structures deposited by the multiplex electrospinner. Guided, intricate designs may be useful when depositing a fiber mat that is functionalized by the configuration used, or where the deposition surface is in a complex configuration

Simultaneous and independent control over electrode voltage and sinusoidal wave inputs of the multiplex electrospinner enables design of intricate structures that could be used for a variety of applications where the deposition surface/s is/are in complex configurations. In Fig. 8, the point of the strongest electrostatic force was moved between electrodes using sinusoidal inputs in a pattern that resulted in formation of looped ring structures.

### Response time and time constant minimization of the multiplex ES system

The multiplex ES system output exhibited a slight lag in response time and decreased amplitude as compared to the input signal (Fig. 4) due to the capability of the high-voltage power supplies to dissipate charge during a desired decrease in the electric field strength. The speed at which the system can respond to changes in the control signal is fundamental to controlling the location of nanofiber deposition; and therefore, minimization of the system time response would result in enhanced control over material morphologies. To understand how the response time of the multiplex system could be reduced, the high voltage power supplies were modeled as charge pumps. In the multiplex system, the high voltage power supplies contain internal capacitors, that cannot dissipate charge instantaneously. The charge pump characteristics of the high voltage power supply has essentially an inertia such that the rate of charge build-up defines the maximum rate that charge can be dissipated. While the rising portion of the input sinusoid signal occurred in equal time for rising and falling portions of the wave (0.368 s for both rising and falling), the output signal exhibited approximately 2X increase in timing for the falling portion of the wave (0.368 s for rising, 0.633 s for falling). As the input frequency is increased, the time discrepancy between rising and falling output signals increases.

**Fig. 9:**
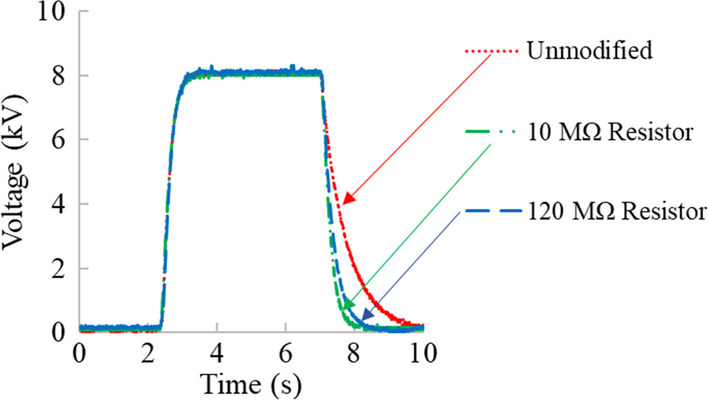
Time response data collected from oscilloscope for the multiplex ES system. Using resistors added in parallel with the high voltage power supplies, the falling time constant that corresponded to charge dissipation of the power supplies was reduced. This reduction in response time of the high voltage power supply to the input signal improves control over fiber deposition and material/device morphology

To decrease the time constant of the multiplex ES system, parallel resistance between the high voltage electrodes and ground were added. Given that the additional resistance is less than that of the unmodified system resistance when placed in parallel, the total resistance of the system decreased significantly with the resistances used. The minimum resistances used were chosen so the maximum current rating of the high voltage power supplies was not exceeded. To determine the time constant of the multiplex ES system, ES was performed initially with a 0 kV input before an input of 20 kV was then supplied to the system. After a steady state output was reached, the system was again given an input of 0 kV. For an increasing signal (the rising portion of the sinusoid), the time constant (τ) is defined as the time it takes the system to go from 0 input voltage to 1–1/e ≈ 63.2% of its final asymptotic value. For a decreasing system (the falling portion of the sinusoid), the time constant is defined as the time it takes the system to decay to 1/e ≈ 36.8% of its initial value. Following determination of the time constants of the multiplex system, resistor banks were added in parallel with the high voltage power supplies, and the rising and falling time constants were again measured. For this work, resistor banks of 10, 50 100, 120 MΩ were used. When using resistors between 10 AND 100 MΩ, the falling time constant was reduced by approximately 63% in all cases (Fig. 9). At 120 MΩ resistance, however, the falling time constant began to increase, and the reduction in time constant was only 51%. This is due to the current rating of the high voltage power supply. Adding resistance in parallel with the system decreases the overall resistance, and subsequently increases the amount of current the system will allow. This increase in current results in improved response times and control over deposition. Between 100 and 120 MΩ, it is assumed that the minimum resistance of the system was reached, and therefore, the 120 MΩ resistor no longer improved the falling time constant response. Figure 8 shows measured voltage used to characterize the time constant when 10 and 120 MΩ resistors were added in parallel with the high voltage power supplies.

In Table 1, the rising and falling time constants of the multiplex system are listed, along with that for the 10 and 120 MΩ resistance:

**Table 1:** Resistors were used to reduce the overall resistance of the system in order to improve response time and control over deposition. When using 10–100 MΩ resistors, the decrease in falling time constant was 63%. When the 120 MΩ resistor was used, the minimum resistance of the system was exceeded, and therefore, the reduction in time constant was not as significant

	Rising time constant (s)	Falling time constant (s)	Reduction in falling time constant (%)
Unmodified response	0.28	0.68	N/A
10 MΩ	0.28	0.25	63%
120 MΩ	0.28	0.33	51%

## Conclusions

Precise control over fiber deposition during ES enables novel device design and promotes new applications and capabilities of the polymer materials produced. In this work, an ES system was fabricated to contain four regular electrodes, each controlled by an independent power supply to create what we term multiplex ES. Use of sinusoidal control signals, amplified by high-voltage power supplies, enabled modifying the electrostatic field strength according to electrode voltage, allowing the user to precisely control fiber deposition and mesoscale structure. Using electrode geometry and separation distances within the multiplex ES system, we were able to determine an analytic model for the electrostatic force acting on a fiber, shown in Eq. 6. Device configuration, material properties, and applied voltage are important in determining the electrostatic force. COMSOL Multiphysics® software was also used as a visualization tool to show the electrostatic field governed by the multiplex ES system and showed that when high-voltage inputs were supplied to specific electrodes, the corresponding electrode voltage in the model matched as predicted.

Less stochastic fiber deposition during multiplex ES enabled creation of a woven fiber mat. Use of sinusoidal inputs moved the high-voltage signal from one electrode to another, producing a woven polymer fabric. In another demonstration, without altering the electrodes, voltage inputs that move from one electrode to the next in a circular pattern produced torus structures. Outer diameter of the torus was found, through parametric analysis, to be rather insensitive to the frequency used during deposition, and inner diameter was found to be inversely related to frequency, resulting in overall thickness of the torus increasing with frequency.

The multiplex ES system exhibited a slight lag in response time as compared to the input signal used to guide fiber deposition. This lag was due to the capability of the high-voltage power supplies to dissipate charge. Because the response rate of the system is fundamental to controlling the location of nanofiber deposition, minimization of the electrical time response was investigated using resistors placed in parallel with the high voltage power supplies and ground. When using resistors between 10 to 100 MΩ, the falling time constant was reduced by approximately 63% in all cases. At 120 MΩ resistance, however, the falling time constant began to increase, and the reduction in time constant was only 51% due to the current rating of the high voltage power supply. It is assumed that the minimum resistance of the system was reached between 100 and 120 MΩ, and therefore, the 120 MΩ resistor no longer improved the falling time constant response.

Multiplex ES has been demonstrated and used to create woven fiber-based mats whereby independent, simultaneous control of high-voltage power supplies, corresponding electrodes, and improved response times resulted in highly-controlled fiber deposition and material morphologies.

## Data Availability

The data relevant to this article is not in a repository. Data included in this article is all presented with exception of the exact LabView program written to control the four electrodes used in multiplex ES. Such a program is proprietary but can easily be replicated by an expert in the field based on the information given in this article.
